# Prevalence, Predictors and Decompressive Laparotomy in Abdominal Compartment Syndrome in Patients Requiring Extracorporeal Membrane Oxygenation

**DOI:** 10.3390/jcm14030855

**Published:** 2025-01-28

**Authors:** Matthias Lubnow, Chiara T. Koch, Maximilian V. Malfertheiner, Maik Foltan, Alois Philipp, Dirk Lunz, Hans J. Schlitt, Frank Brennfleck, Barbara Dietl, Okka W. Hamer, Andrea Stadlbauer, Christof Schmid, Florian Zeman, Thomas Müller, Christoph Fisser

**Affiliations:** 1 Department of Internal Medicine II, University Medical Center Regensburg, 93053 Regensburg, Germany; matthias.lubnow@ukr.de (M.L.); thomas.mueller@ukr.de (T.M.); 2 Center for Pulmonary Medicine, Hospital Donaustauf, 93093 Donaustauf, Germany; maximilian.malfertheiner@klinik-donaustauf.de; 3 Department of Cardiothoracic Surgery, University Medical Center Regensburg, 93053 Regensburg, Germany; maik.foltan@ukr.de (M.F.); alois.philipp@ukr.de (A.P.); andrea.stadlbauer@ukr.de (A.S.); christof.schmid@ukr.de (C.S.); 4 Department of Anesthesiology, University Medical Center Regensburg, 93053 Regensburg, Germany; dirk.lunz@ukr.de; 5 Department of Surgery, University Medical Center Regensburg, 93053 Regensburg, Germany; hans.schlitt@ukr.de; 6 Department of Surgery, Main-Kinzig-Kliniken Gelnhausen, 63571 Gelnhausen, Germany; frank.brennfleck@helios-gesundheit.de; 7 Department of Radiotherapy, University Medical Center Regensburg, 93053 Regensburg, Germany; barbara.dietl@ukr.de; 8 Department of Radiology, University Medical Center Regensburg, 93053 Regensburg, Germany; okka.hamer@ukr.de; 9 Department of Radiology, Hospital Donaustauf, 93093 Donaustauf, Germany; 10 Center for Clinical Studies, University Medical Center Regensburg, 93053 Regensburg, Germany; florian.zeman@ukr.de

**Keywords:** ECMO, abdominal compartment syndrome, ECLS, decompressive laparotomy, prevalence

## Abstract

**Background:** Critically ill patients requiring extracorporeal membrane oxygenation (ECMO) have several risk factors to suffer from abdominal compartment syndrome (ACS). Little is known about this subgroup. The aim of this study was to investigate the prevalence and associated factors for ACS in patients requiring ECMO to assess the effect of decompressive laparotomy (DL) and the impact on mortality. **Methods:** This retrospective observational study analyzed adult patients requiring ECMO in four intensive care units at the University Medical Center Regensburg between 01/2010 and 06/2020. Patients with clinically suspected ACS were screened by measuring intra-abdominal pressure (IAP) with the trans-bladder technique. ACS was defined as IAP > 20 mmHg and survival was defined as successful discharge from hospital. **Results:** The prevalence of ACS in non-ECMO ICU patients was 0.8% (291/36,795) and 2.9% (47/1643) in ECMO patients. In the subgroup of resuscitated ECMO patients, ACS was present in 4.2% (32/766). Procalcitonin was associated with ACS. ECMO patients with ACS receiving DL were significantly more ill compared to those without DL (SOFA score at ICU admission 18 [15; 20], vs. 16 [13; 17], *p* = 0.048). DL decreased IAP and significantly improved ventilation; vasopressor and lactate stabilized within 24 hours. Survival was comparable between the DL and the non-DL groups (11% [1/9] vs. 14% [1/7], *p* = 1.000). **Conclusions:** ECMO patients are at high risk of developing ACS, even more so for resuscitated patients. This and high procalcitonin may be taken into consideration when screening for ACS. Decompressive laparotomy did improve respiratory compliance and stabilized hemodynamic parameters with low rates of complication. Even though patients that received DL were significantly more ill, the mortality rates were not higher.

## 1. Introduction

After the era of fluid resuscitation, little has been published on abdominal compartment syndrome (ACS). No data have been collected regarding a subgroup of patients requiring intensive care. The number of patients requiring extracorporeal membrane oxygenation (ECMO) is steadily increasing as we learn more about the technology and its management [1]. Although ECMO therapy is a potentially life-saving intervention, the procedure itself may cause potentially life-threatening events such as major bleeding [2,3] or thromboembolism [4,5]. Rare events such as abdominal compartment syndrome (ACS) are shown to occur during ECMO in both adults [6] and children [7].

ACS is defined as intra-abdominal pressure (IAP) > 20 mmHg measured with the trans-bladder technique [8]. In critically ill non-ECMO patients, the prevalence of intra-abdominal hypertension (IAH, IAP ≥ 12 mmHg) is >50% [9] and that of ACS is 3–6% [10,11,12]. IAH is associated with renal failure [13], multi-organ failure [14], increased length of stay at the intensive care unit (ICU) [14], and increased mortality [8].

In non-ECMO patients, several risk factors for ACS have been proposed, for instance, diminished abdominal wall compliance, increased intra-luminal contents, capillary leakage, or fluid resuscitation [8]. Each of these conditions may also occur in patients who require ECMO for severe organ failure and are especially often present in patients who have undergone resuscitation. ACS may be prevented and conservatively treated by inducing deep analgo-sedation and neuromuscular blockade, restrictive fluid balance, and evacuating the intra-abdominal contents. However, if these conservative treatments fail, decompressive laparotomy (DL) as an invasive procedure is an option. The goal of DL is to decrease IAH, improve perfusion, and prevent organ deterioration. Clinically major factors in the decision to perform DL are based on the presence of severe ventilatory or other organ dysfunctions created by the mechanical pressure on the lung or hypoperfusion of abdominal organs. DL instantaneously decreases IAH and improves visceral perfusion and ventilation [15]. But little is known if the risk of causing further damage and complications is worth the benefit in ECMO patients who are at substantial risk of bleeding and other complications. To date, data on ACS in patients requiring ECMO are limited to case reports and small case series [6]. Due to the presence of coagulopathy and the associated risk of bleeding in ECMO patients, many ECMO centers are hesitant to perform DL in this population. Nevertheless, the benefits of DL in these patients remain uncertain and therefore need to be studied.

As the number of ECMO patients continues to rise, this study aims to investigate the prevalence of ACS in ECMO patients, identify potential risk factors, and assess whether the benefits of DL outweigh the added risks in this vulnerable population.

## 2. Material and Methods

### 2.1. Study Subjects

This analysis included all patients aged ≥18 years of the University Medical Center Regensburg who had required ECMO between January 2010 and June 2020. We excluded patients below 18 and pregnant patients, patients with incomplete data availability, as well as patients not reaching the diagnostic requirements for ACS.

Further details of the indication for ECMO have been published previously [4,16,17]. This study was conducted according to the Declaration of Helsinki on Good Clinical Practice. The requirement of individual patient consent and the necessity of approval for the data report were waived by the local Ethics Committee (approval code: 20-2114-104, approval date: 18 November 2020) because of the design of this study and the data’s collection from routine care. Patient data, such as demographics, biochemistry, ventilatory and hemodynamic parameters, resuscitation, sequential organ failure assessment (SOFA) score, and applied fluids, were extracted from the electronic inhouse patient data management system. Patients that are addressed as resuscitated underwent resuscitation with consecutive cannulation or extracorporeal cardiopulmonary resuscitation. Neurologic outcome was defined according to the cerebral performance score (CPC) [18] as good (CPC ≤ 2) or poor (CPC > 2), and a successful outcome was discharge from hospital.

### 2.2. ECMO Management

Patients with respiratory failure were mainly cannulated via the right femoral vein (drainage) and the right internal jugular vein (return). Patients with severe cardiac failure were cannulated uni- or bilaterally via the femoral vein and the femoral artery. Patients who could not be weaned from cardiopulmonary bypass after cardiac surgery were cannulated centrally. Adaptions and combinations of veno-venous (VV) and veno-arterial (VA) configurations were used as necessary. The aPTT was aimed to be kept between 40 and 50 s for VV ECMO and 50–60 for VA ECMO. Several different ECMO systems were chosen depending on availability and patient-specific needs, as published previously [4].

### 2.3. Abdominal Compartment Syndrome

All patients underwent a two-step screening assessment for ACS: in the case of a positive clinical assessment (tense abdominal distension or deteriorating renal function, hemodynamics, or ventilation) or sings of ACS in abdominal imaging (PAR, round belly sign [19,20], intra-abdominal pressure (IAP) was measured with the trans-bladder technique [8]. IAH was graded as I (IAP 12–15 mmHg), II (IAP 16–20 mmHg), III (IAP 21–25 mmHg), or IV (IAP > 25 mmHg). ACS was defined according to the current consensus definition of the World Society of the Abdominal Compartment Syndrome as intra-abdominal pressure >20 mmHg and newly developed organ failure [8]. Abdominal perfusion pressure was calculated as mean arterial pressure minus IAP.

### 2.4. Conservative and Surgical Treatment of Abdominal Compartment Syndrome

All patients with IAH were conservatively treated with negative fluid balancing or renal replacement therapy. Laxative measures were used early on to promote bowel movements and evacuate the enteric passage. The decision to perform DL was made by experienced senior surgeons based on the presence of ventilatory and organ dysfunction, the trajectory of lactate as a surrogate for measuring anaerobic metabolism, and the severity of IAH [21]. The presence of ventilatory and other organ dysfunction was assessed; the trajectory of lactate and IAP was used as a surrogate for measuring anaerobic metabolism and the severity of IAH [21].

If conservative treatment was continued, a refractory differential diagnostic was used to rule out other causes of deterioration. After careful case-by-case decision-making depending on individual comorbidities, a decision to perform DL was made with the attending ICU physician and surgeons.

DL was performed in a standardized manner at the bedside or in the operating theater by using a midline incision from the xiphoid process to the pubis. From 2010 to 2018, Barker’s vacuum packing technique was used, and from 2019 onwards, negative pressure therapy with a visceral protection layer was used (3M™ AbThera™ Open Abdomen Negative Pressure Therapy; 3M Company, St. Paul, MN, USA). Final closure of the open abdomen was at the discretion of the surgeon with respect to further treatment and prognosis with or without the implantation of a mesh. The hemodynamic and ventilatory parameters before and after DL were assessed.

### 2.5. Statistics

All quantitative data are expressed as median (interquartile range) and were compared with the Mann–Whitney U-test. Nominal variables were compared between groups using the Chi-squared test of independence or Fisher’s exact test depending on the sample size. Univariable logistic regression models were conducted to analyze the association of clinically relevant variables with ACS. Non-correlated parameters with *p* < 0.05 in the univariable analysis were eligible for multivariable analysis. This model included the following parameters: SOFA score, temperature, lactate, fibrinogen, and procalcitonin. The model was controlled for baseline characteristics such as age, sex, and body mass index. Odds ratios and corresponding 95% confidence intervals are presented as effect estimates for the logistic regression models. All reported *p*-values were two-sided, and a *p*-value of ≤0.05 was considered statistically significant. Data entry and calculation were performed using Microsoft EXCEL365 ProPlus (Microsoft, Redmond, WA, USA) and IBM SPSS Statistic software version 25.0 (SPSS Inc. Chicago, IL, USA).

## 3. Results

### 3.1. Prevalence of Abdominal Compartment Syndrome in ECMO Patients

Between January 2010 and June 2020, 38.438 patients were treated at an ICU of the University Medical Center Regensburg: 36.795 patients without ECMO, 944 with VA- ECMO, and 699 with VV-ECMO. The main indication for VA-ECMO was cardiopulmonary resuscitation (43%) and for VV-ECMO bacterial pneumonia (37%) (Table S1). ACS was diagnosed in 0.8% (291/36.795) of patients in the non-ECMO group and 2.9% (47/1643) in the ECMO group (Figure S1). In comparison to non-ECMO patients with ACS, ECMO patients with ACS were younger and had a higher SOFA score at admission but comparable maximal IAP (28 [24; 30] vs. 30 [25; 30], *p* = 0.825; Table S2).

Within the ECMO group, those requiring VA-ECMO had a higher prevalence of ACS than those with VV-ECMO (3.3% (31/944) vs. 2.3% (16/699)). In the subgroup of resuscitated ECMO patients, including VA and VV ECMO patients, the prevalence of ACS was 4.2% (32/766).

### 3.2. Factors Associated with Abdominal Compartment Syndrome in ECMO Patients

In comparison to ECMO patients without ACS (*n* = 1596), ECMO patients with ACS (*n* = 47) had been more often resuscitated and had a higher SOFA score with more impaired hemodynamics, higher inflammatory parameters, more deranged coagulation, lower temperature, and bytrendlower paO_2_/FiO_2_ despite comparable ventilation and circulatory support (Table 1). Procalcitonin was associated with ACS (OR 1.024, 95-CI [1.006; 1.042], *p* = 0.008) in the multivariable analysis (Table 2).

### 3.3. Decompressive Laparotomy and Outcome for Abdominal Compartment Syndrome in ECMO Patients

A total of 62% of patients received laxatives measures, 19% renal replacement therapy, and 6% negative fluid balancing. Other conservative therapies were not applied in = specific patient cases (Table S3).

DL was performed in 55% (26/47) of ECMO patients with ACS who had shown higher IAP, a higher SOFA score, less respiratory system compliance, lower temperature, increasing renal failure, and by trend increasing liver failure and pancreatitis (Table 3 and Table S5). Other than that, no differences between the DL and the non-DL groups were found (Table S5).

DLs were performed at the ICU (81%) or in the operating room (19%), both with a very low rate of surgical complications (Table S4). Transfusions of packed red blood cells and platelets per day on ECMO were comparable between the DL and the non-DL groups (Table 3).

**Figure 1:**
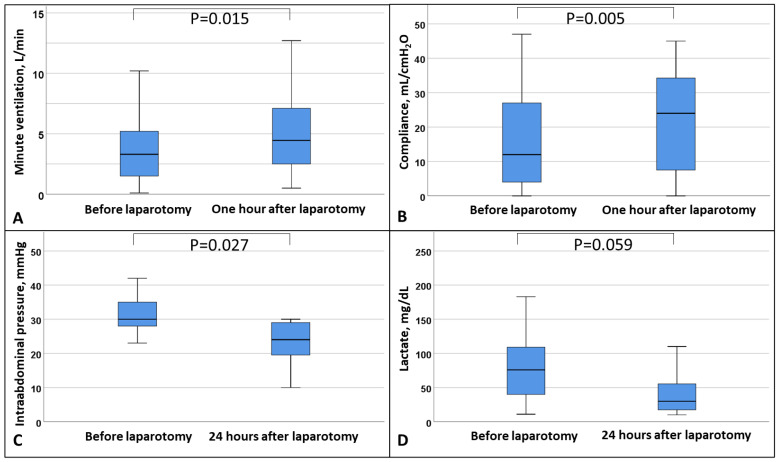
Box plot of (**A**) minute ventilation, (**B**) respiratory system compliance, (**C**) intra-abdominal pressure, and (**D**) lactate before and after decompressive laparotomy. Data are expressed as median, minimum, maximum, 25. percentile, and 75. percentile.

After DL ventilation improved (Figure 1), IAP was lowered and resulted in lower lactate and vasopressor levels by trend, while ECMO blood flow was stable (Table 4). The trajectories of hemodynamics and ECMO flow within 48 h were similar between the DL and the non-DL groups (Table S5). Notably, the decrease in IAP within 24 h showed a comparable trend between the two groups, but patients in the DL group had higher initial IAP (DL: −8 [−20; −6] vs. no DL: −7 [−10; −2], *p* = 0.863).

**Figure 2:**
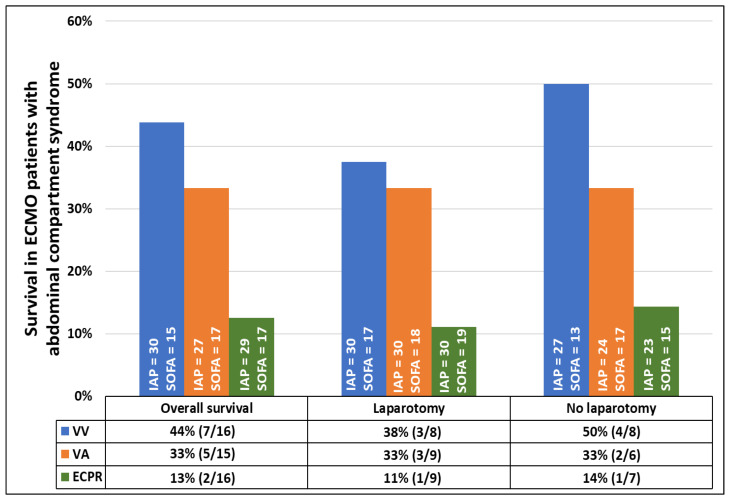
Survival rate of patients with abdominal compartment syndrome requiring veno-venous or veno-arterial extracorporeal membrane oxygenation, or extracorporeal cardiopulmonary resuscitation according to decompressive laparotomy. Data are presented in percentages. Intra-abdominal pressure and sequential organ failure assessment are presented as median values. All *p*-values comparing survival rates within each group of patients with and without decompressive laparotomy are >0.05.

**Figure 3:**
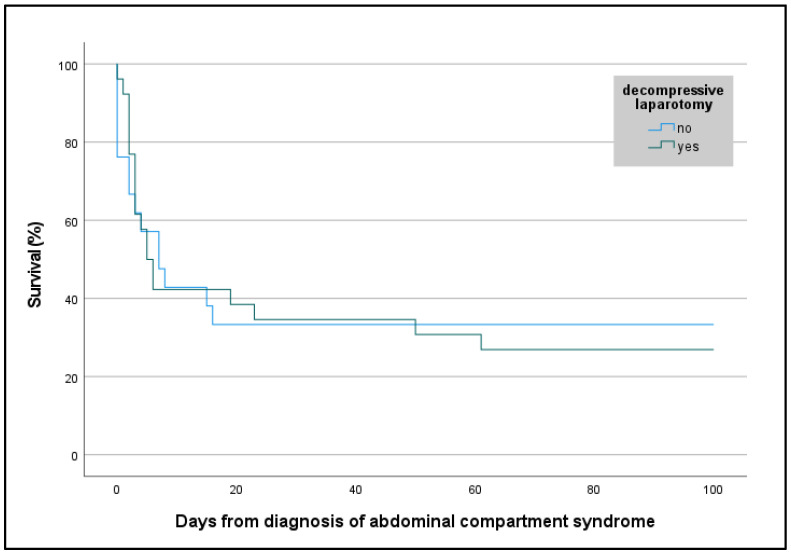
Kaplan–Meier curve with survival rate of patients with abdominal compartment syndrome requiring veno-venous or veno-arterial extracorporeal membrane oxygenation, or extracorporeal cardiopulmonary resuscitation according to decompressive laparotomy. Survival is presented in percentages. Days from diagnosis of abdominal compartment syndrome were assessed retrospectively.

Survival to hospital discharge was lower in ECMO patients with ACS than in ECMO patients without ACS (30% [14/47] vs. 51% [818/1596], *p* = 0.004), but the neurologic outcome was good in both groups (ECMO ACS: 100% [14/14] vs. ECMO non-ACS: 98% [802/817], *p* = 1.000). In ECMO patients with ACS, even though the DL group was significantly more ill (SOFA score 16 [13; 17] vs. 18 [15; 20], *p* = 0.048), survival was similar between the DL and the non-DL groups (27% [7/26] vs. 33% [7/21], *p* = 0.633), irrespective of the ECMO configuration (Figure 2 and Figure 3). Resuscitated ECMO patients with ACS had worse survival than those without resuscitation (7/32 [22%] vs. 7/15 [47%], *p* = 0.088).

## 4. Discussion

This study provides new insights into the prevalence and associated factors of ACS in patients requiring ECMO. Furthermore, it provides new information about the safety and outcome of ECMO patients who have undergone laparotomy as a treatment option.

Firstly, the prevalence of ACS was significantly higher in ECMO patients compared to intensive care patients without ECMO. Resuscitated ECMO patients had the highest prevalence of ACS.

Secondly, ECMO patients with ACS had higher disease severity and lower survival than ECMO patients without ACS. ACS was associated with higher procalcitonin levels.

Thirdly, DL decreased IAP, improved respiration, and by trend decreased lactate and vasopressor dependance. A very low rate of surgical complications was observed, without raising transfusion requirements. Even though patients who were treated with DL were significantly more ill, presenting with higher initial IAP and markers for organ failure, their survival did not differ from the conservatively treated patients with lower IAP and markers for organ failure.

### 4.1. Prevalence of Abdominal Compartment Syndrome in ECMO Patients

Most ECMO patients have several risk factors for ACS, such as mechanical ventilation with positive end-expiratory pressure > 10 cmH_2_O, pneumonia, shock, and coagulopathy [8]. Consequently, the prevalence of ACS in ECMO patients was more than three times higher than that in non-ECMO patients. Resuscitated ECMO patients were more likely to suffer from ACS in the current study. The higher prevalence of ACS in resuscitated patients was anticipated because of the generally higher disease severity in VA ECMO populations [17]. Other studies using systematic screening for ACS by measuring IAP every 8–12 h yielded a prevalence of 3–6% in non-ECMO patients [10,11,12]. The prevalence in our ECMO cohort was lower, which may be due to the applied two-step screening algorithm in this analysis and a lower number of surgical patients than in the two largest prospective screening studies for ACS in non-ECMO cohorts [10,12]. The current data confirm that not only patients with surgical diagnoses but also patients with other severe diseases that necessitate ECMO are significantly affected by ACS [8].

### 4.2. Factors Associated with Abdominal Compartment Syndrome in ECMO Patients

In patients with ACS, high IAP generally leads to reduced abdominal organ perfusion [15] and as a consequence of bowel ischemia or other abdominal organ failure [8,13]. Other authors reported an independent association of lactate with IAP [12]. Yet, in the multivariable analysis, only procalcitonin as a marker of bacterial infection was associated with ACS. Diebel et al. suggested that reduced mucosal blood flow might contribute to the development of ACS by promoting bacterial translocation from the gut [22], which could be reflected by elevated PCT levels [23]. The implication of this is that ACS should be considered as a differential diagnosis in patients with elevated procalcitonin levels, particularly when no clear source of infection is identified. Further research is needed to validate the potential of procalcitonin as a marker for ACS for clinical use.

### 4.3. Decompressive Laparotomy and Outcome for Abdominal Compartment Syndrome in ECMO Patients

To date, the decision to perform DL in non-ECMO and ECMO patients with ACS is made on an individual basis by the treating physician and the surgeon [21,24,25,26]. In the current study, patients with higher IAP and progressive organ dysfunction such as renal impairment and reduced respiratory system compliance were considered for DL, with AH being the best independent predictor of ICU mortality [11]. In addition, DL in non-ECMO patients is known to improve ventilatory settings to enable more protective ventilation [15]. In this study of ECMO patients, within one hour after DL, lower respiratory pressures were applied to the lung because of improved respiratory system compliance, which increased tidal volume and minute ventilation and by trend decreased lactate and vasopressor dependance.

Despite substantial improvements in surgical techniques, complication and mortality rates in surgical patients undergoing DL are still relatively high [15]. It remains unclear whether these rates also apply to ECMO patients with ACS, who less often present with a surgical abdominal diagnosis but have more pronounced overall critical illness. ECMO patients are generally more often affected by bleeding complications [5]. Even with these risk factors present, the rate of complications in ECMO patients receiving DL in this study was low. Transfusion requirements were not raised. Our data confirm that IAP was reduced but not normalized 24 h after DL, which underlines the importance of continued IAP monitoring after DL.

In the DL group, one wound healing disorder and one spleen laceration with bleeding occurred. However, the transfusion requirements for neither PRBCs nor platelets were significantly elevated in the DL group, nor was the survival between the DL and the non-DL groups different.

Survival in the DL group did not differ from the conservatively treated group. The similar survival rates and neurologic outcome of ECMO patients with and without DL are remarkable, particularly when considering the more extensive critical illness at baseline in patients requiring DL (e.g., higher IAP and a higher SOFA score).

A considerable number of complications commonly affect patients who are resuscitated. Therefore, it is not surprising that this sub-population of ECMO patients had the highest prevalence of ACS and lower survival than the non-resuscitated cohort of ECMO patients with ACS. In our cohort, however, the neurological outcome was good, whether or not DL was required to treat ACS.

### 4.4. Limitations

This retrospective single-center study was conducted by staff with considerable experience in the management of ECMO. Still, the prevalence of ACS may be slightly underdiagnosed because of the application of a widely clinically used two-step screening procedure in this analysis compared to studies using systematic routine trans-bladder screening. However, clinical signs of distended abdomen, which were used as a first screening step in this study, are the strongest independent predictor of ACS [12]. These results cannot be extrapolated to patients with IAH ≤ 20 mmHg. Outcome analyses have to be considered in light of the limited number of patients. Beside demographics, only limited data were available for the non-ECMO cohort. This study is limited by potential biases due to its retrospective design and the single-center setting. Future prospective studies should involve larger and multicenter cohorts to identify patients who may benefit the most from DL.

## 5. Conclusions

ECMO patients are at high risk of developing ACS. Resuscitated patients are even more susceptible. High procalcitonin can be taken into consideration when screening. In this study, decompressive laparotomy did improve respiratory compliance immediately and stabilized hemodynamic parameters, with low rates of complications observed. Even though patients that received DL were significantly more ill, the mortality rates were not higher.

## Figures and Tables

**Table 1:** Baseline patient characteristics before extracorporeal membrane oxygenation.

Variables	No Abdominal Compartment Syndrome (n = 1596)	Abdominal Compartment Syndrome (n = 47)	*p*-Value
Age, years	57 [47; 66]	58 [48; 67]	0.978
Male sex	1111 (70%)	35 (74%)	0.475
BMI, kg/m^2^	27.0 [24.2; 31.1]	27.7 [23.9; 35.6]	0.331
Temperature, °C	35.0 [33.5; 36.2]	33.9 [32.5; 35.4]	**0.002**
ECMO mode, VA	913 (57%)	31 (66%)	0.232
SOFA	14 [11; 17]	17 [14; 19]	**<0.001**
Resuscitation	734 (46%)	32 (68%)	**0.003**
Renal replacement therapy	283 (18%)	13 (28%)	0.081
Mean arterial pressure, mmHg	61 [45; 70]	55 [40; 65]	**0.032**
Norepinephrine, µg/kg/min	0.30 [0.12; 0.60]	0.51 [0.00; 0.97]	0.080
paO_2_/FiO_2_, mmHg	77 [60; 127]	70 [56; 100]	0.086
Minute ventilation, L/min	9.0 [6.0; 11.4]	8.0 [5.0; 11.5]	0.298
Tidal volume, mL	460 [417; 560]	450 [427; 556]	0.249
Respiratory rate, /min	18 [13; 23]	17 [11; 22]	0.259
Peak inspiratory pressure, cmH_2_O	30 [24; 35]	30 [25; 38]	0.225
Positive end-expiratory pressure, cmH_2_O	12 [9; 15]	13 [9; 15]	0.394
pH	7.22 [7.14; 7.32]	7.14 [7.01; 7.22]	**<0.001**
Lactate, mg/dL	50 [18; 99]	94 [56; 157]	**<0.001**
Activated partial thromboplastin time, s	45 [35; 70]	72 [42; 120]	**<0.001**
D-Dimer, mg/L	7 [3; 20]	14 [6; 34]	**0.001**
Fibrinogen, mg/dL	349 [215; 540]	185 [109; 354]	**<0.001**
Platelets, 10^9^/L	176 [116; 251]	167 [92;212]	0.073
C-reactive protein, mg/L	57 [11; 165]	34.5 [3; 132]	0.108
Procalcitonin, µg/L	3.5 [1.0; 13.2]	47.0 [14.0; 93.0]	**<0.001**
Interleukin-6, pg/mL	401 [112; 1703]	1673 [367; 18300]	**0.001**
Interleukin-8, pg/mL	107 [40; 346]	407 [57; 2154]	**0.006**
Lactate dehydrogenase, U/L	438 [278; 744]	511 [347; 809]	0.262
Aspartate aminotransferase, U/L	98 [44; 288]	149 [69; 406]	0.080
Plasma free hemoglobin, mg/L	101 [46; 273]	298 [127; 599]	**<0.001**

Data are expressed as n (%), median [25. percentile; 75. percentile]; ECMO: extracorporeal membrane oxygenation; VA: veno-arterial; SOFA: sequential organ failure assessment; significant *p*-values (*p* < 0.05) are marked in bold.

**Table 2:** Associated factors for abdominal compartment syndrome—multivariable logistic regression model.

Variables	OR (95% CI)	*p*-Value
SOFA	1.040 (0.807; 1.340)	0.764
Temperature, °C	0.813 (0.604; 1.096)	0.175
Lactate, mg/dL	1.006 (0.994; 1.018)	0.339
Fibrinogen, mg/dL	1.001 (0.998; 1.005)	0.429
Procalcitonin, µg/L	1.024 (1.006; 1.042)	**0.008**

All parameters were assessed within one hour before extracorporeal membrane oxygenation. Model was controlled for age, sex, and body mass index. Significant *p*-values (*p* < 0.05) are marked in bold.

**Table 3:** Patient characteristics at diagnosis of abdominal compartment syndrome in patients receiving extracorporeal membrane oxygenation (n = 47), stratified according to decompressive laparotomy.

Variable	N	No Decompressive Laparotomy (n = 21)	N	Decompressive Laparotomy (n = 26)	*p*-Value
Intra-abdominal pressure, mmHg					**0.001**
III (21–25 mmHg)	12	12 (57%)	3	3 (12%)	
IV (>25 mmHg)	9	9 (43%)	23	23 (88%)	
Age, years	21	59 [48; 66]	26	58 [46; 67]	0.923
Male sex	21	17 (81%)	26	18 (69%)	0.360
BMI, kg/m^2^	21	29.4 [26.0; 37.0]	25	27.5 [23.7; 36.9]	0.434
SOFA at ICU admission	14	16 [13; 17]	21	18 [15; 20]	**0.048**
Temperature, °C	14	36.8 [33.9; 37.1]	25	34.8 [33.3; 35.5]	**0.022**
Abdominal perfusion pressure, mmHg	21	40 [33; 53]	23	36 [25; 49]	0.169
Mean arterial pressure, mmHg	21	68 [60; 78]	23	67 [55; 79]	0.851
Epinephrine, µg/kg/min	20	0.05 [0.00; 0.11]	23	0.10 [0.00; 0.17]	0.163
Norepinephrine, µg/kg/min	21	0.14 [0.00; 0.31]	22	0.07 [0.00; 0.19]	0.595
Resuscitation	21	15 (71%)	26	21 (81%)	0.452
pO2/FiO2, mmHg	16	153 [85; 296]	18	133 [85; 208]	0.551
Minute ventilation, L/min	20	3.9 [2.9; 5.3]	21	3.4 [1.6; 6.1]	0.382
Tidal volume, mL	20	335.5 [207.8; 401.5]	21	214.0 [73.5; 322.0]	**0.039**
Respiratory rate, /min	20	13 [10; 17]	18	16 [14; 20]	0.051
Peak inspiratory pressure, cmH_2_O	20	27.0 [23.0; 30.8]	21	30.0 [27.5; 34.0]	**0.036**
Positive end-expiratory pressure, cmH_2_O	19	14 (11.8; 20]	21	15.0 [13.7; 20.0]	0.486
Respiratory system compliance, mL/cmH_2_O	18	34.0 [10.8; 38.8]	19	19.5 [3.0; 30.0]	**0.046**
Acute liver failure	21	2 (10%)	26	9 (35%)	0.081
Acute renal failure	21	9 (43%)	26	22 (85%)	**0.003**
Net volume until ACS, mL	21	2134 [74; 4799]	16	3867 [1898; 7219]	0.066
pH	21	7.40 [7.30; 7.40]	22	7.30 [7.20; 7.48]	0.570
Lactate, mg/dL	21	99 [21; 137]	23	88 [43; 152]	0.365
ECMO flow, L/min	21	3.3 [3; 3.5]	23	3.5 [3.0; 3.9]	0.424
Days on ECMO	21	5 [2; 8]	26	4 [2; 7]	0.644
PRBCs per day on ECMO support	21	1.0 [0.2; 2.3]	26	1.6 [0.4; 3.5]	0.371
Platelets transfusions per day on ECMO support	21	0 [0; 0]	26	0 [0; 0]	0.422

Data are expressed as n (%), median [25. percentile; 75. percentile]; ACS: abdominal compartment syndrome; ECMO: extracorporeal membrane oxygenation; PRBCs: packed red blood cells; significant *p*-values (*p* < 0.05) are marked in bold. All values were assessed at the time of abdominal compartment diagnosis until otherwise stated.

**Table 4:** Comparison of ventilatory and hemodynamic parameters in patients requiring extracorporeal membrane oxygenation with abdominal compartment syndrome before and after decompressive laparotomy (*n* = 26).

Variable	n	Before Decompressive Laparotomy	n	After Decompressive Laparotomy	*p*-Value
Intra-abdominal pressure, mmHg	26	30 [28;35]	11	24 [19; 29]	**0.027**
Minute ventilation, L/min	21	3.3 [1.4; 5.5]	22	4.5 [2.5; 7.2]	**0.015**
Tidal volume, mL	21	227 [113; 349]	22	341 [183; 444]	**0.006**
Respiratory Rate, /min	21	15 [14; 19]	22	15 [13; 19]	0.894
Peak inspiratory pressure, cmH_2_O	21	31.0 [29.0; 35.0]	21	30.0 [28.0; 33.0]	0.139
Positive end-expiratory pressure, cmH_2_O	21	18.6 [15.7; 20.3]	21	18.0 [15.4; 20.1]	0.440
Driving pressure, cmH_2_O	20	14.4 [11.0; 15.4]	20	12.7 [10.0; 15.4]	0.221
Compliance, mL/cmH_2_O	19	12.0 [3.0; 29.0]	19	24.0 [7.0; 34.5]	**0.005**
ECMO blood flow, L/min	25	3.5 [3.0; 3.9]	25	3.5 [3.0; 4.0]	0.373
Epinephrine, µg/kg/min	26	0.07 [0.00; 0.12]	22	0.00 [0.00; 0.07] ^a^	0.344
Norepinephrine, µg/kg/min	26	0.08 [0.00; 0.19]	22	0.00 [0.00; 0.13] ^a^	0.109
Mean arterial pressure, mmHg	24	70 [60; 94]	22	68 [59; 75] ^a^	0.126
Lactate, mg/dL	12	76 [38; 119]	24	30 [19; 78] ^a^	0.059
Urine volume, mL/h	24	30 [11; 140]	24	36 [13; 177]	0.173

Data are expressed as n (%), median [25. percentile; 75. percentile]. Values were assessed at the time of decompressive laparotomy and one hour after decompressive laparotomy. ^a^ Values were assessed 24 h after decompressive laparotomy. Significant *p*-values (*p* < 0.05) are marked in bold.

## Data Availability

The datasets used and analyzed during the current study are available from the corresponding author on reasonable request.

## Supplementary Materials

The following supporting information can be downloaded at: https://www.mdpi.com/article/10.3390/jcm14030855/s1. Figure S1: Flowchart of the observational study evaluating abdominal compartment syndrome in critically ill patients with and without extracorporeal membrane oxygenation at the University Medical Center Regensburg. ECMO: extracorporeal membrane oxygenation; ACS: abdominal compartment syndrome; Table S1: Indication for extracorporeal membrane oxygenation; Table S2: Comparison of baseline characteristics of patients with abdominal compartment syndrome with and without extracorporeal membrane oxygenation; Table S3: Preventive measures in patients with abdominal compartment syndrome requiring extracorporeal membrane oxygenation, stratified according to treatment with decompressive laparotomy; Table S4: Details on decompressive laparotomy in patients with abdominal compartment syndrome who required extracorporeal membrane oxygenation; Table S5: Trajectories of intraabdominal pressure, extracorporeal membrane oxygenation flow, and hemodynamics within 48 hours after treatment of abdominal compartment syndrome according to decompressive laparotomy.

## Abbreviations

ACS	abdominal compartment syndrome
BMI	body mass index
CPC	cerebral performance category
DL	decompressive laparotomy
ECMO	extracorporeal membrane oxygenation
IAH	intra-abdominal hypertension
IAP	intra-abdominal pressure
ICU	intensive care unit
SOFA	sequential organ failure assessment
VA	veno-arterial
VV	Veno-venous
