# Supplementary material for: Prevalence, Predictors and Decompressive Laparotomy in Abdominal Compartment Syndrome in Patients Requiring Extracorporeal Membrane Oxygenation
Source: J Clin Med. 2025 Jan 28;14(3):855. doi: 10.3390/jcm14030855 (PMC11818648; doi:10.3390/jcm14030855)
Supplement: Supplementary file 1 [file jcm-14-00855-s001.zip › jcm-3431709-supplementary.pdf]

Supplementary Materials:

This appendix has been provided by the authors to give readers additional information about this study.

This is a supplement to the following study:

Prevalence, Predictors and Decompressive Laparotomy in Abdominal Compartment Syndrome in Patients Requiring Extracorporeal Membrane Oxygenation

Matthias Lubnow [1,†], Chiara T. Koch [1,*,†], Maximilian V. Malfertheiner [2], Maik Foltan [3], Alois Philipp [3], Dirk Lunz [4], Hans J. Schlitt [5], Frank Brennfleck [6], Barbara Dietl [7], Okka W. Hamer [8,9], Andrea Stadlbauer [3], Christof Schmid [3], Florian Zeman [10], Thomas Müller [1] and Christoph Fisser [1,*]

1 Department of Internal Medicine II, University Medical Center Regensburg, 93053 Regensburg, Germany; matthias.lubnow@ukr.de (M.L.); thomas.mueller@ukr.de (T.M.)
2 Center for Pulmonary Medicine, Hospital Donaustauf, 93093 Donaustauf, Germany; maximilian.malfertheiner@klinik-donaustauf.de
3 Department of Cardiothoracic Surgery, University Medical Center Regensburg, 93053 Regensburg, Germany; maik.foltan@ukr.de (M.F.); alois.philipp@ukr.de (A.P.); andrea.stadlbauer@ukr.de (A.S.); christof.schmid@ukr.de (C.S.)
4 Department of Anesthesiology, University Medical Center Regensburg, 93053 Regensburg, Germany; dirk.lunz@ukr.de
5 Department of Surgery, University Medical Center Regensburg, 93053 Regensburg, Germany; hans.schlitt@ukr.de
6 Department of Surgery, Main-Kinzig-Kliniken Gelnhausen, 63571 Gelnhausen, Germany; frank.brennfleck@helios-gesundheit.de
7 Department of Radiotherapy, University Medical Center Regensburg, 93053 Regensburg, Germany; barbara.dietl@ukr.de
8 Department of Radiology, University Medical Center Regensburg, 93053 Regensburg, Germany; okka.hamer@ukr.de
9 Department of Radiology, Hospital Donaustauf, 93093 Donaustauf, Germany
10 Center for Clinical Studies, University Medical Center Regensburg ,93053 Regensburg, Germany; florian.zeman@ukr.de
* Correspondence: chiara.koch@ukr.de (C.T.K.); christoph.fisser@ukr.de (C.F.)
† These authors contributed equally to this work.

Figure S1. Flowchart of the observational study evaluating abdominal compartment syndrome in critically ill patients with and without extracorporeal membrane oxygenation at the University Medical Center Regensburg. ECMO: extracorporeal membrane oxygenation; ACS: abdominal compartment syndrome.

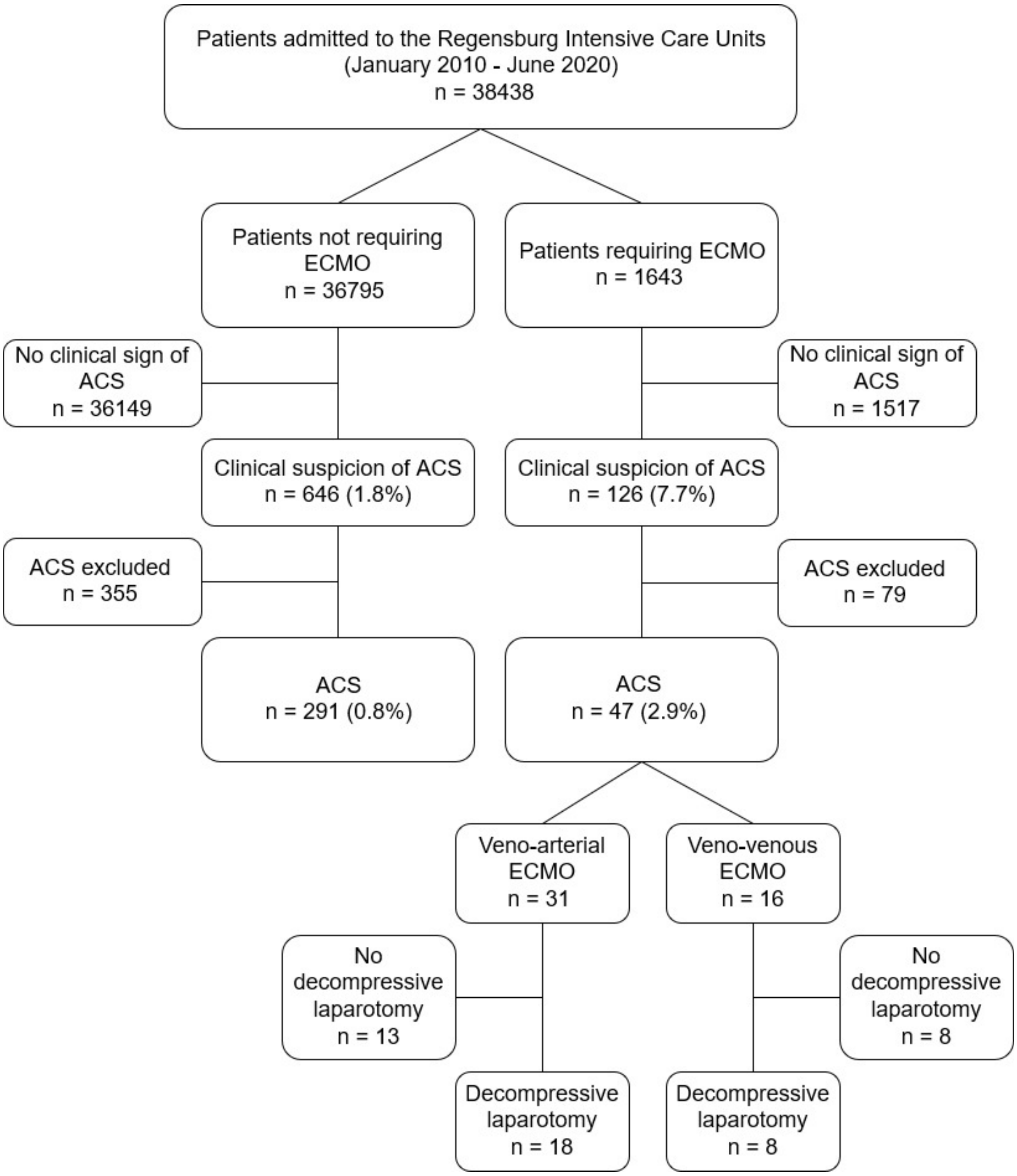

Table S1. Indication for extracorporeal membrane oxygenation

Variable	Veno-arterial (n = 944)	Veno-venous (n = 699)
Ongoing resuscitation (ECPR), n (%)	402 (43%)	
Cardiac failure during coronary intervention, n (%)	57 (6%)	
Low cardiac output, n (%)	216 (23%)	
Low cardiac output after resuscitation within the past 12 h, n (%)	171 (18%)	
Not weanable after cardiopulmonary bypass, n (%)	98 (10%)	
ARDS group I (pulmonary), n (%)		421 (60%)
ARDS group II (extrapulmonary), n (%)		142 (20%)
ARDS group III (traumatic), n (%)		53 (8%)
ARDS/ALF group IV (miscellaneous), n (%)		83 (12%)

Data are expressed as n (%); ECPR: extracorporeal cardiopulmonary resuscitation; ARDS: acute respiratory distress syndrome; ALF: acute lung failure; miscellaneous includes drowning, pulmonary embolism, cystic fibrosis, bridge to transplant, etc.

Table S2. Comparison of baseline characteristics of patients with abdominal compartment syndrome with and without extracorporeal membrane oxygenation

**Variable**	**ACS without ECMO (n = 291)**	**ACS and ECMO (n = 47)**	***p*-Value**
Age, years	62 [51; 72]	58 [47; 66]	**0.022**
Male sex	194 (67%)	35 (74%)	0.176
Body mass index, kg/m² (admission)	27.8 [24.7; 31.9]	27.8 [24.2; 37.0]	0.335
SOFA (on admission)	12 [10; 14][a]	17 [14; 19][b]	**<0.001**
Maximum intraabdominal pressure, mmHg	30 [25; 30]	28 [24; 30]	0.825

Data are expressed as n (%), median (25. percentile; 75. percentile); ECMO: extracorporeal membrane oxygenation; SOFA: sequential organ failure assessment; [a]n=58; [b]n=36; significant p-values (p <0.05) are marked in bold.

Table S3. Preventive measures in patients with abdominal compartment syndrome requiring extracorporeal membrane oxygenation, stratified according to treatment with decompressive laparotomy

**Variable**	**Overall (n = 47)**	**No decompressive laparotomy (n = 21)**	**Decompressive laparotomy (n = 26)**	***p*-Value**
Laxative measurements (other than neostigmine)	20 (43%)	12 (57%)	8 (32%)	0.087
Neostigmine	9 (19%)	6 (29%)	3 (13%)	0.267
Defecation	9 (19%)	5 (26%)	4 (20%)	0.716
Renal replacement therapy	9 (19%)	4 (19%)	5 (20%)	1.000
Negative fluid balancing the day before diagnosis of ACS	3 (6%)	2 (10%)	1 (4%)	0.579
Evacuation of the enteric passage (e.g., colonoscopy)	0 (0%)	0 (0%)	0 (0%)	-
Ascites punction	0 (0%)	0 (0%)	0 (0%)	-

Data are expressed as n (%). ACS: abdominal compartment syndrome; all parameters were assessed within 24 hours before diagnosis of ACS.

Table S4. Details on decompressive laparotomy in patients with abdominal compartment syndrome who required extracorporeal membrane oxygenation

Decompressive laparotomy	(n=26)
Location of decompressive laparotomy	
Intensive care unit	21 (81%)
Operating theater	5 (19%)
Days until abdominal closure	8 [6; 14]
Intra-abdominal packing	18 (69%)
Amount of used abdominal linen	1.5 [0; 2.5]
Bowl resection	5 (19%)
Negative pressure wound therapy	
Barker's vacuum packing technique	22 (85%)
Negative pressure therapy with visceral protection layer	4 (15%)
Number of re-operations	1 [0; 2]
Complications	2 (8%)
Wound healing disorder	1 (4%)
Spleen laceration, bleeding	1 (4%)
Packed red blood cells transfusion after decompressive laparotomy per day on ECMO support [b]	1.0 [0.0; 2.3]
Implanted abdominal mesh	18 (69%)

Data are expressed as n (%), median [25. percentile; 75. percentile]; ECMO: extracorporeal membrane oxygenation; [a] assessed in n = 9 patients; [b] only days after decompressive laparotomy were included.

Table S5. Trajectories of intraabdominal pressure, extracorporeal membrane oxygenation flow, and hemodynamics within 48 hours after treatment of abdominal compartment syndrome according to decompressive laparotomy.

**Variables**	**No decompressive laparotomy (n = 21[a])**	**Decompressive laparotomy (n = 26[b])**	***p*-Value**
Maximum intraabdominal pressure, mmHg	24 [22; 29]	30 [28; 35]	**0.002**
Intraabdominal pressure after 24 hours, mmHg	17 [14; 24]	24 [19; 29]	0.204
Intraabdominal pressure after 48 hours, mmHg	16 [9; 17]	18 [13; 22]	0.280
ECMO blood flow, L/min	3.3 [3.0; 3.5]	3.5 [3; 3.9]	0.435
ECMO blood flow after 12 hours, L/min	3.3 [2.7; 3.6]	3.2 [2.7; 4]	0.728
ECMO blood flow after 48 hours, L/min	3.0 [2.7; 3.8]	3.2 [2.6; 4]	0.621
Epinephrine, µg/kg/min	0.05 [0.00; 0.11]	0.10 [0.00; 0.17]	0.163
Epinephrine after 24 hours, µg/kg/min	0.00 [0.00; 0.02]	0.00 [0.00; 0.08]	0.338
Epinephrine after 48 hours, µg/kg/min	0.00 [0.00; 0.03]	0 [0.00; 0.05]	0.389
Norepinephrine hours, µg/kg/min	0.14 [0.00; 0.31]	0.07 [0.00; 0.19]	0.595
Norepinephrine after 24 hours, µg/kg/min	0.00 [0.00; 0.10]	0.02 [0.00; 0.07]	0.564
Norepinephrine after 48 hours, µg/kg/min	0.00 [0.00; 0.05]	0.02 [0.00; 0.26]	0.073
Mean arterial pressure, mmHg	68 [60; 78]	70 [60; 94]	0.419
Mean arterial pressure after 24 hours, mmHg	71 [62; 79]	68 [59; 75]	0.665
Mean arterial pressure, after 48 hours, mmHg	68 [60; 83]	71 [62; 75]	0.934

Data are expressed as n (%), median [25. percentile; 75. percentile]. Values were assessed at [a] diagnosis of abdominal compartment syndrome and [b] the time of decompressive laparotomy. Significant p-values (p <0.05) are marked in bold.
